# Psychometric Validation of the CLN2 Quality of Life Questionnaire in Participants with CLN2 Disease Treated with Cerliponase Alfa

**DOI:** 10.3390/healthcare12222229

**Published:** 2024-11-08

**Authors:** Christina Due, Jennifer Quinn, Paul Gissen, Angela Schulz, Nicola Specchio, Emily de los Reyes, Thomas Butt

**Affiliations:** 1 BioMarin International Ltd., London WC1A 2SL, UK; 2 Debiopharm Inc., 1006 Lausanne, Switzerland; 3 National Institute for Health Research Great Ormond Street Hospital Biomedical Research Centre, University College London, London WC1N 1EH, UK; paul.gissen@gosh.nhs.uk; 4 Department of Pediatrics, University Hospital Hamburg-Eppendorf, 20246 Hamburg, Germany; 5 German Center for Child and Adolescent Health (DZKJ), Partner Site Hamburg, 20251 Hamburg, Germany; 6 Neurology, Epilepsy and Movement Disorders Unit, Bambino Gesù Childrens Hospital, IRCCS, European Reference Network EpiCARE, 00165 Rome, Italy; nicola.specchio@gmail.com; 7 University Hospitals KU Leuven, 3000 Leuven, Belgium; 8 Nationwide Children’s Hospital, Columbus, OH 43205, USA

**Keywords:** CLN2, quality of life, CLN2 QoL, psychometric testing, clinically important difference

## Abstract

Objectives: This study evaluated the psychometric properties of the ceroid lipofuscinosis type 2 Quality of Life (CLN2 QoL) questionnaire. Methods: Data from children with CLN2 disease aged 3–16 years receiving cerliponase alfa in the BMN 190-201 and BMN 190-202 clinical studies, collected via purposive sampling, were used to assess convergent and divergent validity, internal consistency and reliability. The clinically important difference (CID) was estimated with distribution- and anchor-based methods. Descriptive and inferential statistical analyses were conducted using IBM SPSS. Results: CLN2 QoL data of 22 participants were analysed. Ceiling effects were observed in 22 items (35% threshold); no floor effects were observed. Internal consistency analysis showed good reliability (Cronbach’s alpha and Omega reliability >0.7) for four domains at study completion; only one domain had good reliability at baseline. All domains had good test–retest reliability (correlation >0.5) except Feeding With G-Tube and Seizures. Convergent and divergent correlation analysis showed moderate-strong correlations (>0.4) between PedsQL and CLN2 QoL total scores, between the Pediatric Quality of Life Inventory (PedsQL) total score and most CLN2 QoL domains at baseline, and between CLN2 QoL total score and most PedsQL domains at week 97. Known groups validity showed a significant difference in means for the Behaviour domain (*p* = 0.05) for reasons that could not be clarified. CID was 6.79–12.94 for domains; total score CID was 6.91 using distribution-based and 6.13–13.05 using anchor-based methods. Conclusions: This study is the first to validate the CLN2 QoL and to estimate the CID of this instrument in CLN2 patients. Our results show good validity and reliability of this tool.

## 1. Introduction

Neuronal ceroid lipofuscinosis type 2 (CLN2) disease (also known as Jansky–Bielschowsky disease) is one type of a group of genetic disorders known as Batten disease [1]. CLN2 is an autosomal recessive, pediatric neurodegenerative disease that is characterised by deficiency of the lysosomal serine protease tripeptidyl peptidase-1 (TPP1) caused by mutations in the *CLN2* gene. The incidence of CLN2 varies between different regions. Estimates of CLN2 live births per 100,000 vary in different regions, from 0.15 (Portugal) to 9 (Newfoundland); a total of 14,000 people in the world are estimated to have CLN2 [2,3,4]. Affected individuals experience speech delay, seizures, ataxia, loss of vision and rapid cognitive and motor decline, but presentation can vary between patients [5]. The onset of symptoms usually occurs between the ages of 2 and 4 years and includes seizures and delayed language. A rapid decline in motor, language, cognitive and visual function follows at the age of 4 years. If untreated, death will occur in early adolescence [6,7,8,9,10].

Cerliponase alfa (Brineura^®^) is the only treatment currently available for CLN2 and was approved in the USA and Europe in 2017. It is an enzyme replacement therapy that is designed to help restore TPP1 enzyme activity; it is administered intraventricularly every 2 weeks [11]. The long-term efficacy, safety, tolerability and pharmacokinetics of cerliponase alfa in patients with CLN2 disease were evaluated in two clinical trials: BMN 190-201 (ClinicalTrials.gov number: NCT01907087) and its extension study, BMN 190-202 (ClinicalTrials.gov number: NCT02485899). Twenty-four participants were enrolled, with 23 patients enrolling in the extension trial. Enrolled patients were between the ages of 3 and 16 years; the mean age was 5 years. Nine males and 15 females were included in the study. The results from the clinical trials showed significant reduction in the rate of decline in motor and language function compared with historical controls [11,12]. Additionally, recent single-centre studies showed slowing of brain volume loss and progression of movement disorders in treated children [13,14]. The pivotal cerliponase alfa clinical trials evaluated the disease severity using disease-specific scales including the clinician-reported 12-point Adapted Clinical Rating Scale (12-point scale amended from the Hamburg late infantile neuronal ceroid lipofuscinoses [LINCL] Scale) and 6-point Clinical Rating Scale ML (6-point scale including Motor and Language domains of the Adapted Clinical Rating Scale), and the parent/caregiver-reported Pediatric Quality of Life Inventory (PedsQL^TM^) [11,15] and CLN2 Quality of Life (QoL) questionnaires. The CLN2 QoL questionnaire is a parent/caregiver-reported outcome measure that has been developed to measure the QoL of patients with CLN2; it was developed based on the evaluation of family feedback from two focus groups.

Recent improvements in early diagnosis of CLN2 have allowed for early treatment initiation, thus reducing the rate of rapid decline of motor and language functions compared with historical untreated controls [16,17,18]. To address the need for a reliable tool to measure changes in health-related quality of life (HRQoL) and support use of the CLN2 QoL questionnaire in clinical trials, this post hoc analysis of clinical trial data aimed to provide an evaluation of the psychometric properties and estimate the clinically important difference (CID) of the questionnaire (Supplementary File S1) in patients treated with cerliponase alfa.

## 2. Methods

### 2.1. Population

The data used for the analysis were derived via purposive sampling from the open-label, single-arm, phase 1/2 BMN 190-201 study and its ongoing extension BMN 190-202, including 24 children aged 3 to 16 years who had been diagnosed with CLN2 disease and had a combined score of 3 to 6 on the Clinical Rating Scale ML [11,12].

### 2.2. Assessments

The CLN2 QoL questionnaire, the PedsQL, the PedsQL family impact modules, the Adapted Clinical Rating Scale and the Clinical Rating Scale ML were assessed longitudinally throughout both studies. For BMN 190-201, the tools were administered at baseline, start of the stable dose period, every 12 weeks thereafter, and at study completion/early termination visit. For BMN 190-202, they were administered every 24 weeks and at study completion/early termination visit.

### 2.3. Instruments Used in the Psychometric Analysis

The CLN2 QoL instrument, which was developed by BioMarin, has a recall period of 1 month. It has 28 items that cover six domains: Seizures (six items), Feeding (no G-Tube) (four items), Feeding (With G-Tube) (three items), Sleep (five items), Behaviour (six items) and Daily Activities (four items). Items are scored on a 5-point Likert scale ranging from 0 (“never”), 1 (“almost never”), 2 (“sometimes”), 3 (“often”) to 4 (“almost always”). Each item was reverse scored and linearly transformed to a 0–100 scale: 0 = 100, 1 = 75, 2 = 50, 3 = 25, 4 = 0. A total mean score was calculated as the sum of all items over the total number of items answered on all domains. Domain scores were calculated for each of the six domains as means of the items within the domains. If more than 50% of the items in a domain were missing, the mean domain score was not computed.

The PedsQL Generic Core Scales are designed to measure QoL in children and adolescents. The assessments are brief, practical and developmentally appropriate. The tool consists of five domains that include the measurement of physical, emotional, social and school functioning, and has both child self-reported and parent proxy-report versions (https://www.pedsql.org/ accessed on 31 October 2024). Each of the items within a scale is scored as 0 (never a problem), 1 (almost never a problem), 2 (sometimes a problem), 3 (often a problem), to 4 (almost always a problem). Items are then reverse scored and linearly transformed to a 0–100 scale as follows: 0 = 100, 1 = 75, 2 = 50, 3 = 25 and 4 = 0, so that higher scores indicate better health-related QoL (HRQoL). The instrument is responsive to clinical change over time. The four parent-reported versions cover the ages of 1–12 months, 13–24 months, 2–4 years and 5–7 years, and include questions regarding physical, emotional and social functioning, with school functioning where applicable.

Disease progression was measured using disease-specific scales, including the Adapted Clinical Rating Scale and the Clinical Rating Scale ML [15,19,20]. The Adapted Clinical Rating Scale is based on the Hamburg LINCL Scale and includes four categories including Motor, Language, Visual scores and Seizures. Each function is scored between 0 and 3, with a total score ranging from 0 to 12. The Clinical Rating Scale ML consists of the Motor and Language domains of the Adapted Clinical Rating Scale [11]. Each function is scored from 0 to 3, with a rating of 3 indicating normal function, and a total score ranging from 0 to 6. A higher score represents better function [15]. Time until the first unreversed 2-point decline in the combined score was the primary outcome of the clinical trial [11].

### 2.4. Statistical Analysis

The psychometric validity of the CLN2 QoL was evaluated by measuring the item facility, reliability and the construct validity. In addition, the CID was determined. A summary of the analyses is provided in Table 1. The data were tested for normality and data distribution to confirm they met the assumptions for using parametric tests. All analyses were conducted on data from patients in BMN 190-201 using IBM SPSS Version 29 for descriptive (percentage, mean, SD, reliability test) and inferential analysis (for example, Pearson Correlation).

### 2.5. Reliability

Floor and ceiling effects were calculated at item level using baseline data. CLN2 QoL items are scored from 0 to 4 and reverse scored, where a score of 4 signifies the worst possible outcome (floor) and 0 signifies the best possible outcome (ceiling). The score distribution at minimum and maximum limits was assessed at a threshold of 35%, with additional thresholds at 20% and 50% also considered.

The CLN2 QoL was also tested for internal consistency, which aims to assess how closely related a set of items measure the same concept [21]. This was measured by calculating Cronbach’s alpha and Omega reliability, where a score of ≥0.7 represents good internal consistency. Due to lack of data for one domain (Feeding With G-Tube) at baseline, two additional timepoints (week 97 and study completion) were also selected. Cronbach’s alpha was calculated for all items, items within domains and domains within the structure, due to the low number of participants in this study.

Test–retest reliability aims to measure the reliability of a scale by assessing the closeness of agreement between two timepoints in stable patients. For the present analysis, intraclass correlation coefficients (ICC) and Pearson’s correlation coefficients were calculated at baseline and Stable dose week 13 for domain scores and the total score. A timepoint closer to baseline (Stable dose week 1) could not be selected due to missing data. Therefore, moderate to strong correlations of ≥0.5 between baseline and week 13 were considered acceptable.

### 2.6. Validity

Convergent validity assesses the degree of a relationship between an existing measure and the instrument of interest. Similarly, divergent validity means that domains of both instruments do not correlate too strongly, as they measure different concepts. In this study, convergent and divergent validity were determined by calculating Pearson’s correlation coefficients between the CLN2-QoL and PedsQL scores at baseline, with additional analyses conducted for week 97. Moderate correlations were defined as a correlation coefficient of 0.4–5.9 and strong correlations as a correlation coefficient of 0.6–1.0.

Known groups validity is the ability for a scale to distinguish between different participant subgroups. In the BMN 190-201 and BMN 190-202 studies, only patients with CLN2 were recruited. Therefore, a comparison between CLN2 and non-CLN2 patients could not be made. The reference measure used to assess CLN2 severity was the Adapted Clinical Rating Scale. Groups were created based on the Adapted Clinical Rating Scale scores: 4, 6, 7, 8, 9, 10 and 12. One-way ANOVAs were performed to determine whether CLN2 QoL scores differed between subgroups with different Adapted Clinical Rating Scale scores at baseline.

### 2.7. Determination of CID

Distribution-based method and anchor-based methods were used to determine the CID. Distribution-based methods were used to provide support for responder thresholds. This was performed by calculating half the standard deviation (SD) at baseline, as it has been shown by numerous studies that the threshold for clinically relevant changes in HRQoL for chronic conditions is half an SD [22]. Anchor-based methods examine the relationship between the HRQoL measured by the instrument of interest and an independent anchor to determine the mean difference in change. Due to the small sample size, the CID was calculated for different time frames, i.e., changes in CLN2 QoL total scores from baseline to week 97, from baseline to study completion, and from week 97 to study completion, and compared between anchor categories of the Clinical Rating Scale ML (+1, 0, −1 and −2 change in Motor and Language, 0–6).

## 3. Results

### 3.1. Patient Characteristics

Descriptive statistics of the CLN2 QoL, PedsQL, Adapted Clinical Rating Scale and Clinical Rating Scale ML scores of patients included in the analysis at baseline are presented in Supplementary File S2, Tables S1–S3.

### 3.2. Reliability

Floor and ceiling effects are shown in Table 2. Results for Feeding With G-Tube are not shown due to lack of data. At 35%, ceiling effects were observed for 22 items; there were no floor effects observed (Supplementary File S2, Table S4). At 20%, ceiling effects were observed for 24 items, and floor effects were observed for 2 items. At 50%, ceiling effects were observed for 13 items, but no floor effects were observed.

For the internal consistency analysis, the Cronbach’s alpha coefficients at item level for baseline, week 97 and study completion all showed good internal consistency (0.83, 0.86 and 0.83, respectively) (Table 3). However, when separating items by domain, only Sleep had good internal consistency at baseline (0.83) and Sleep and Seizures were the only domains that had good internal consistency (0.79, 0.90, respectively) at week 97. At study completion, Sleep, Feeding No G-Tube, Seizures and Feeding With G-Tube all had good internal consistency (0.80, 0.74, 0.82 and 0.79, respectively). The Omega reliability data were aligned with the Cronbach’s alpha results.

For test–retest reliability, a summary of the ICC and Pearson’s correlation is shown in Table 4. A good test–retest reliability assumed the score was moderately to highly correlated for the same domain over a short time interval (baseline and week 13). The domain Feeding No G-Tube had a moderate correlation with the ICC (0.420) and a poor Pearson’s correlation (0.266). Neither of these correlations was significant. For Seizures, there was a low positive correlation with ICC (0.301) and a poor Pearson’s correlation (0.179). Neither of these correlations was significant. For Feeding With G-Tube, the two measurements analysed were for week 97 and week 121 due to no data at baseline and week 13. The ICC showed a significant strong reliability of 0.882, and there was a significant strong Pearson’s correlation of 0.822. Sleep had a significant strong ICC reliability of 0.847 and a significant strong Pearson’s correlation of 0.776. Behaviour had a significant moderate ICC reliability of 0.640 and a significant moderate Pearson’s correlation of 0.478. Daily Activities had a significant moderate ICC of 0.570 and a significant moderate Pearson’s correlation of 0.443. The total score had a significant strong ICC reliability of 0.743 (*p* = 0.002) and a strong Pearson’s correlation of 0.591 (*p* = 0.005). This indicates that, overall, the CLN2 QoL tool had good test–retest reliability.

### 3.3. Validity

#### 3.3.1. Convergent and Divergent Validity

Results for convergent validity of the CLN2 QoL and PedsQL are shown in Table 5 for baseline and in Table 6 for week 97. Moderate to strong correlations (>0.4) were expected for most domains with the exception of the PedsQL School Functioning and Emotional Functioning domains. For the baseline results, there were no or weak correlations between Emotional Functioning, Social Functioning and School Functioning. Physical Functioning had moderate correlations with Feeding No G-Tube, Seizures and total score. PedsQL Psychosocial Functioning had moderate correlations with CLN2 QoL Seizures, Sleep, Behaviour and total score. The PedsQL total score had moderate correlations with all CLN2 domains and total score with the exception of Daily Activities.

The week 97 results differ from baseline results. CLN2 Feeding No G-Tube had moderate correlations with all PedsQL domains with the exception of School Functioning. CLN2 Seizures, Feeding With G-Tube and Behaviour had no or weak correlations with PedsQL domains and total score. CLN2 Sleep was moderately correlated with Physical Functioning, Emotional Functioning and total score. CLN2 Daily Activities was correlated with all PedsQL domains. CLN2 total score was correlated with all PedsQL domains except School Functioning. The total scores for PedsQL and CLN2 QoL were moderately to highly correlated; this was observed at both baseline and week 97. These correlations support a level of agreement between the two tools. However, they are not measuring the same construct as the PedsQL is a generic QoL tool for a pediatric population that has not been validated in CLN2 patients.

#### 3.3.2. Known Groups Validity

**Figure 1:**
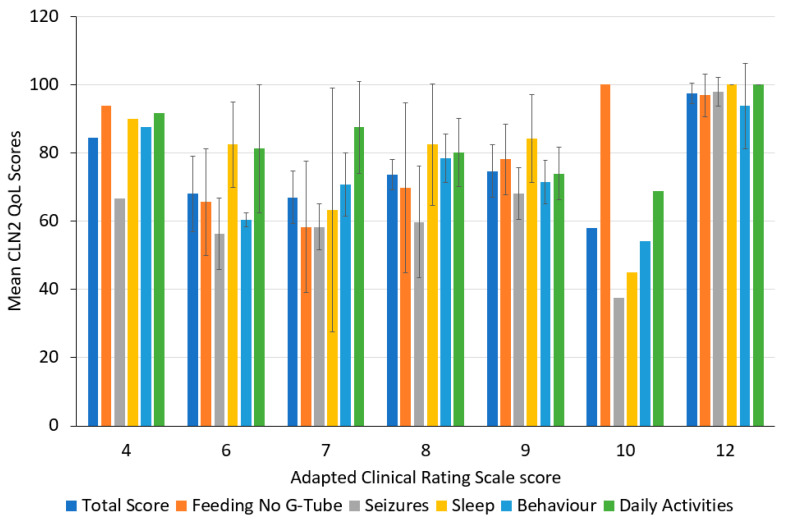
Mean CLN2 Quality of Life (CLN2 QoL) scores grouped by Adapted Clinical Rating Scale score at baseline. Error bars represent standard deviations.

Results of the ANOVA comparing mean CLN2 QoL scores for the different Adapted Clinical Rating Scale groups are shown in Figure 1. There was a significant difference in means for Behaviour (*p* = 0.05); however, no differences were observed for the other domains. A post-hoc Tukey test could not be performed because Clinical Rating Scale ML groups 4 and 10 had only one participant in each group.

### 3.4. Clinically Important Difference

CIDs estimated using a distribution-based method are shown in Table 7. The CID ranged from 6.79 to 12.94 for the domains and was 6.91 for the total score.

Results of the anchor-based CID analyses are shown in Table 8, Table 9 and Table 10. A score change of 1 in the Clinical Rating Scale ML (Language and Motor function) was used as the anchor. For participants that had a Clinical Rating Scale ML score change of 1 (from +1, 0, −1 and −2 change), the mean magnitude of changes from baseline to week 97 for the CLN2 QoL were 28.2, 2.2, 4.9 and 15.3, respectively. A comparison of groups indicated that a score change of >13.1 would correspond to a meaningful change in CLN2 QoL total score. When the one subject who showed an increase in the Clinical Rating Scale ML score (+28.2) was excluded from the analysis, the CID estimate decreased to 6.6.

The anchor-based analysis was repeated from baseline to study completion, where the mean magnitude of change for the CLN2 QoL was 4.5 (+1 score change), 5.5 (no change), 3.3 (−1 change) and 18.5 (−2 change). A comparison of groups indicated that a score change of >6.1 corresponds with a meaningful change in CLN2 QoL total score. Due to the patients showing stability over time, the CID estimate was repeated from week 97 to study completion. The mean magnitude of change from week 97 to study completion was 11.9 for subjects whose rating increased, 2.5 for subjects with no change and 8.1 for subjects whose rating scale decreased (−1). A comparison of groups indicated a score change of 7.7.

## 4. Discussion

This study is the first to assess the psychometric properties of the CLN2 QoL questionnaire and to estimate the CID for this instrument. Due to the rarity of CLN2, there are currently no validated tools to assess HRQoL for this disease, underscoring the relevance of this study.

The results of floor and ceiling effects found a high level of ceiling effects at a 35% threshold, but no floor effects were observed. The presence of ceiling effects at baseline may restrict the ability of the item to detect improvement throughout the study. The clinical studies (BMN 190-201 and BMN 190-202) showed that patients treated with cerliponase alfa had a slower rate of decline of disease compared with historical controls; an improvement of disease was not observed.

The Cronbach’s alpha for all items and domains analysed together was >0.70 at the three different timepoints, indicating that items in the questionnaire measure the same concept. However, for items within domains, the Cronbach’s alpha had low values at baseline (except for the Sleep domain), with some improvement observed at study completion. This could be explained by the low number of participants in the trial, as well as missing data. This issue will be covered in more detail under study limitations. Despite the low number of participants, the Sleep domain had a high Cronbach’s alpha across all three timepoints, indicating that it has higher internal consistency than the other domains.

For the analysis of test–retest reliability, moderate to strong correlations were considered to indicate good reliability due to the large time difference between timepoints. Ideally, when conducting test–retest reliability analysis, two close timepoints are selected, as this ensures patient stability that minimises any variation in their condition. In this analysis, narrower timepoints could not be selected due to missing data. Overall, most domains and total score found moderate to high levels of agreement between the two timepoints, indicating test–retest reliability in the CLN2 QoL instrument.

Convergent validity analysis showed that total scores of the CLN2 QoL and the PedsQL were highly correlated with each other, which suggests the two tools are conceptually similar. Although the correlations between domains did differ in some of the predictions, results were largely consistent with expectations, particularly for week 97. The difference in correlations between the two timepoints (baseline and week 97) could be indicative of the low sample size. It should also be noted that the PedsQL has not been validated in CLN2 patients. However, a recent study showed significant correlations between the Clinical Rating Scale ML and the PedsQL [23]. For analysing known groups validity, it was hypothesised that patients who had a higher Adapted Clinical Rating Scale score would have a higher CLN2 QoL score. While the results did show there was a significant difference in means for the Behaviour domain, the results are difficult to interpret as the post-hoc Tukey test could not be performed due to a low sample size, and two of the categories (4 and 10 for Adapted Clinical Rating Scale score) had only one participant in each group. Therefore, it is difficult to draw conclusions from the known groups analysis.

The CID estimated using distribution-based methods at baseline ranged from 6.79 to 12.94 for the different CLN2 QoL domain scores, with the total score CID being 6.91. To supplement this analysis, an anchor-based approach using the Clinical Rating Scale ML (0–6 Language and Motor) as an anchor was also used at baseline, week 97 and study completion. The different timepoints had very different CID estimates: baseline to week 97 had a CID estimate of 13.1, whereas baseline to study completion had an estimate of 6.1, which is similar to the CID estimated using distribution-based methods in this study. The difference between both analyses appeared to be due to the single subject in the baseline to week 97 analysis showing an increase in Clinical Rating Scale ML score between the two timepoints; this patient had an increase in CLN2 QoL score of 28.2. The remaining subjects were equally distributed between the other categories. When the analysis was repeated for these timepoints without the outlier, the CID estimate was reduced to 6.6. Caution should be applied when calculating a clinically meaningful score change, as patients in this trial showed a very slow decline in disease progression. Based on the analysis in this study taken at different timepoints, the CID value is estimated to be between 6.1 and 7.7.

The main limitation of this study is the small sample size, which made some analyses difficult to interpret and did not allow for testing differences in sample characteristics, such as age and sex. Because of this, different timepoints were used in the analyses. The analysis would benefit from a larger sample size; however, due to the ultra-rare disease status of CLN2, this will be very difficult to obtain. The difficulty in achieving a bigger sample size is further highlighted by the accelerated approval of cerliponase alfa that was granted by the EMA and FDA based on the small pivotal sample size [24,25]. In addition, the domain Feeding With G-Tube had very little data across the study, which also complicated some analyses that included this domain. The CLN2 QoL questionnaire was collected by proxy, which could introduce some bias, as has been documented in previous studies [26]. However, due to the young age of the patients and the pronounced decline of disease, a proxy response is the only viable option for collecting patient outcomes.

Future work could include an analysis using item response theory to further evaluate the items’ ability to discriminate. This method has shown promising results for small sample sizes [27,28]. Other additional assessments that may be performed when a bigger dataset is available are exploratory factor analysis, which can uncover hidden patterns and relationships among variables, and confirmatory factor analysis to confirm construct validity [29].

## 5. Conclusions

This study is the first to validate the CLN2 QoL instrument in CLN2 patients and to estimate the CID in this population. Our results demonstrate that the CLN2 QoL tool displays good validity and reliability, supporting its use in clinical trials and clinical practice. This has become even more relevant given the recent improvements in diagnosing these patients, allowing earlier initiation of treatment and better treatment outcomes [16,17,18]. While tools such as the Hamburg LINCL Scale are important for measuring disease progression, QoL questionnaires allow families to convey the day-to-day impact of the disease on their lives and the community in general. The CID analysis established a CID estimated using two different methods, which can help in the interpretation of study results in terms of clinical relevance.

## Figures and Tables

**Table 1:** Summary of psychometric analyses of the CLN2 Quality of Life (CLN2 QoL) questionnaire.

Property	Analysis Period	Definition	Success Criterion
Item facility	Baseline	Floor and ceiling effects	35% (20%, 50% also analysed)
Internal consistency	Baseline, week 97 and study completion	Cronbach’s alpha Omega reliability	≥0.70
Test–retest reliability	Baseline to SD week 13	Pearson’s correlations and ICC	|r| ≥ 0.4
Convergent and divergent validity	Baseline and week 97	Pearson’s correlations	|r| ≥ 0.4
Known groups validity	Baseline	Mean difference between groups (Clinical Rating Scale ML), ANOVA	*p* < 0.05; effect size ≥ 5%
CID with distribution-based methods	Baseline	Half the SD	Estimates reported
CID with anchor-based methods	Baseline to week 97 and study completion; week 97 to study completion	Mean change from baseline in relation to change in anchor groups (Clinical Rating Scale ML)	Estimates reported

CID: clinically important difference; ICC: intraclass correlation coefficient; SD: standard deviation.

**Table 2:** Floor and ceiling effects of the CLN2 Quality of Life (CLN2 QoL) questionnaire.

Domain		Min (Ceiling Effect)				Max (Floor Effect)		
	Range ^1^	20% ^2^	35% ^2^	50% ^2^	Range ^1^	20% ^2^	35% ^2^	50% ^2^
Feeding No G-Tube (4 items)	50.0–72.7%	4 (100%)	4 (100%)	3 (75%)	4.5–22.7%	1 (25%)	0	0
Seizures (6 items)	13.6–45.5%	5 (83.3%)	4 (66.6%)	0	4.5–27.3%	1 (16.7%)	0	0
Feeding With G-Tube (3 items)	n/a							
Sleep (5 items)	59.1–86.4%	5 (100%)	5 (100%)	5 (100%)	4.5–18.2%	0	0	0
Behaviour (6 items)	31.8–86.4%	6 (100%)	5 (83.3%)	2 (33.3%)	4.5–22.7%	1 (16.7%)	0	0
Daily Activities (4 items)	38.1–81.8%	4 (100%)	4 (100%)	3 (75%)	4.5–14.3%	0	0	0

^1^ Percentage of responses in items with the highest vs. lowest ceiling/floor effects; ^2^ Number and % of items in every scale showing ceiling/floor effects for 20%, 35%, 50% thresholds; n/a: not available.

**Table 3:** CLN2 Quality of Life (QoL) Cronbach’s alpha and Omega reliability for all items, items within domains and domains within the structure for baseline, week 97 and study completion.

All items	N	Cronbach’s Alpha	Omega Reliability
Baseline	25	**0.83**	n/a
Week 97	25	**0.86**	n/a
Study completion (BMN 190-202)	24	**0.83**	n/a
**Items within domains**			
**Baseline**			
Seizures	6	0.56	0.62
Feeding No G-Tube	4	0.65	n/a
Sleep	5	**0.83**	**0.84**
Daily Activities	4	0.15	n/a
Behaviour	6	0.20	n/a
Feeding With G-Tube	n/a		n/a
**Week 97**			
Seizures	6	**0.90**	**0.91**
Feeding No G-Tube	4	0.47	0.45
Sleep	5	**0.79**	**0.81**
Daily Activities	4	0.62	0.65
Behaviour	6	0.64	0.53
Feeding With G-Tube	3	0.11	n/a
**Study completion (BMN 190-202)**			
Seizures	6	**0.82**	**0.82**
Feeding No G-Tube	4	**0.74**	**0.74**
Sleep	5	**0.80**	**0.80**
Daily Activities	4	0.53	0.57
Behaviour	6	0.56	0.56
Feeding With G-Tube	3	**0.79**	**0.89**
**Domains within the structure**			
Baseline	5	**0.74**	**0.74**
Week 97	5	**0.71**	**0.68**
Study completion (BMN 190-202)	5	**0.70**	**0.72**

**Cronbach’s Alpha and Omega reliability ≥0.7 are highlighted**; n/a: not available.

**Table 4:** Test–retest reliability: Intraclass correlation coefficients and Pearson’s correlations for the CLN2 Quality of Life (CLN2 QoL) questionnaire.

	Intraclass Correlation Coefficient (95%CI)	Pearson’s Correlation
Feeding No G-Tube baseline and Stable dose week 13	**0.420** (−0.430–0.765)	0.266
Seizures baseline and Stable dose week 13	0.301 (−0.723–0.716)	0.179
Feeding With G-Tube week 97 and week 121	**0.882** (0.156–0.983) *	0.822 *
Sleep baseline and Stable dose week 13	**0.847** (0.622–0.938) *	0.776 *
Behaviour baseline and Stable dose week 13	**0.640** (0.113–0.854) *	0.478 *
Daily Activities baseline and Stable dose week 13	**0.570** (−0.059–0.826) *	0.443 *
Total score baseline and Stable dose week 13	**0.743** (0.365–0.896) *	0.591 *

* Correlation is significant at the 0.05 level (2-tailed). **Correlations > 0.40 are highlighted**.

**Table 5:** Convergent validity between baseline CLN2 Quality of Life (CLN2 QoL) questionnaire and Pediatric Quality of Life Inventory (PedsQL) domain and total scores with Pearson’s correlation.

	PedsQL					
	Physical Functioning	Emotional Functioning	Social Functioning	Psychosocial Functioning	School Functioning	Total Score
CLN2 Feeding No G-Tube	0.478 *	−0.022	0.334	0.259	0.027	0.482 *
CLN2 Seizures	0.434 *	0.267	0.344	0.426 *	0.102	0.550 **
CLN2 Sleep	0.295	0.397	0.208	0.451 *	0.007	0.467 *
CLN2 Behaviour	0.351	0.249	0.068	**0.411**	0.297	0.482 *
CLN2 Daily Activities	0.157	−0.047	−0.189	−0.086	−0.118	0.057
CLN2 Total score	0.484 *	0.295	0.257	0.453 *	0.092	0.599 **

* Correlation is significant at the 0.05 level (2-tailed). ** Correlation is significant at the 0.01 level (2-tailed). **Moderate to strong correlations > 0.40–1.00 are highlighted.**

**Table 6:** Convergent validity between week 97 CLN2 Quality of Life (CLN2 QoL) questionnaire and Pediatric Quality of Life Inventory (PedsQL) domain and total scores with Pearson’s correlation.

	PedsQL					
	Physical Functioning	Emotional Functioning	Social Functioning	Psychosocial Functioning	School Functioning	Total Score
CLN2 Feeding No G-Tube	0.463 *	0.481 *	0.379	0.481 *	0.160	0.551 **
CLN2 Seizures	0.316	0.324	0.290	0.334	0.063	0.381
CLN2 Feeding With G-Tube	0.200	−0.624	−0.178	−0.295	−0.257	−0.070
CLN2 Sleep	0.483 *	0.444 *	0.203	0.374	0.220	0.499 *
CLN2 Behaviour	−0.036	**0.502**	0.200	0.333	−0.063	0.188
CLN2 Daily Activities	0.443 *	0.558 **	0.426 *	0.609 **	0.486 *	0.616 **
CLN2 Total score	0.479 *	0.645 **	**0.405**	0.582 **	0.223	0.622 **

* Correlation is significant at the 0.05 level (2-tailed). ** Correlation is significant at the 0.01 level (2-tailed). **Moderate to strong correlations > 0.40–1.00 are highlighted.**

**Table 7:** Distribution-based clinically important difference (CID) estimates for the CLN2 Quality of Life (CLN2 QoL) questionnaire.

	Baseline Mean	SD	Distribution-Based CID Estimate
Feeding No G-Tube	75.57	22.57	11.28
Seizures	64.20	18.17	9.08
Sleep	79.77	25.89	12.94
Behaviour	73.67	13.57	6.79
Daily Activities	81.72	15.35	7.67
Total score	74.20	13.82	6.91

SD: standard deviation.

**Table 8:** Mean difference in CLN2 Quality of Life (CLN2 QoL) questionnaire total score at baseline and week 97 in subjects with a CLN2 rating scale ML change of 1.

	Subjects with Improvement in Clinical Rating Scale ML (+1)		Subjects with No Change in Clinical Rating Scale ML (0)		Subjects Worsening in Clinical Rating Scale ML (−1)		Subjects Worsening in Clinical Rating Scale ML (−2)	
	N = 1		N = 8		N = 9		N = 5	
Mean magnitude of CLN2 QoL score change	28.2		2.2		5.0		15.3	
Subjects difference in magnitude of change vs. change scores		26.0		2.7		10.5		
Relative magnitude of Clinical Rating Scale ML change								13.1

**Table 9:** Mean difference in CLN2 Quality of Life (CLN2 QoL) questionnaire total score at baseline and study completion in subjects with a CLN2 rating scale ML score of 1.

	Subjects with Improvement in Clinical Rating Scale ML (+1)		Subjects with No Change in Clinical Rating Scale ML (0)		Subjects Worsening in Clinical Rating Scale ML (−1)		Subjects Worsening in Clinical Rating Scale ML (−2)	
	N = 1		N = 8		N = 9		N = 5	
Mean magnitude of CLN2 QoL score change	4.5		5.5		3.3		18.5	
Subjects difference in magnitude of change vs. change scores		1		2.2		15.2		
Relative magnitude of Clinical Rating Scale ML change								6.1

**Table 10:** Mean difference in CLN2 Quality of Life (CLN2 QoL) questionnaire total score at week 97 and study completion in subjects with a CLN2 rating scale ML score of 1.

	Subjects with Improvement in Clinical Rating Scale ML (+2)		Subjects with Improvement in Clinical Rating Scale ML (+1)		Subjects with No Change in Clinical Rating Scale ML (0)		Subjects Worsening in Clinical Rating Scale ML (−1)	
	N = 1		N = 8		N = 9		N = 5	
Mean magnitude of CLN2 QoL score change	20.2		9.1		2.5		8.1	
Subjects difference in magnitude of change vs. change scores		10.9		6.7		5.6		
Relative magnitude of Clinical Rating Scale ML change								7.7

## Data Availability

The de-identified individual participant data that underlie the results reported in this article (including text, tables, figures, Supplementary Materials) will be made available together with the research protocol and data dictionaries for noncommercial, academic purposes. Additional supporting documents may be available upon request. Investigators will be able to request access to these data and supporting documents via a website (www.BioMarin.com accessed on 31 October 2024) beginning 6 months and ending 2 years after publication. Data associated with any ongoing development program will be made available within 6 months after approval of the relevant product. Requests must include a research proposal clarifying how the data will be used, including proposed analysis methodology. Research proposals will be evaluated relative to publicly available criteria available at www.BioMarin.com to determine if access will be given, contingent upon execution of a data access agreement with BioMarin Pharmaceutical Inc.

## Supplementary Materials

The following supporting information can be downloaded at: https://www.mdpi.com/article/10.3390/healthcare12222229/s1, Supplementary File S1: CLN2 Quality of Life Questionnaire; Supplementary File S2: Table S1: Descriptive summary of CLN2 Quality of Life (CLN2 QoL) questionnaire domains at baseline in the BMN 190-201 study, Table S2: Descriptive summary Pediatric Quality of Life Inventory (PedsQL) domains at baseline in the BMN 190-201 study, Table S3: Descriptive summary Clinical Rating Scale ML and Adapted Clinical Rating Scale scores at baseline in the BMN 190-201 study, Table S4: Ceiling and floor effects for individual items in the CLN2 Quality of Life (CLN2 QoL) questionnaire
