# Supplementary material for: Psychometric Validation of the CLN2 Quality of Life Questionnaire in Participants with CLN2 Disease Treated with Cerliponase Alfa
Source: Healthcare (Basel). 2024 Nov 8;12(22):2229. doi: 10.3390/healthcare12222229 (PMC11593549; doi:10.3390/healthcare12222229)
Supplement: Supplementary file 1 [file healthcare-12-02229-s001.zip › Supplementary File S1.pdf]

CLN2 Disease Based Quality of Life

CLN2-QL

Version 1.0

DIRECTIONS

The following questions are to be answered by the parent or caregiver taking care of the child with CLN2 disease. The list is a series of disease-related issues that might be a problem for you, your family and the child with CLN2. Please tell us **how much of a problem** each has been during **the past ONE month** by circling the most accurate score:

0 if it is **never** a problem

1 if it **almost never** a problem

2 if it is **sometimes** a problem

3 if it is **often** a problem

4 if it is **almost always** a problem

There are no right or wrong answers. If you do not understand a question, please ask for help.

BIOMARIN®

CLN2 DISEASE BASED QUALITY OF LIFE ASSESSMENT

Date performed: ___ ___ /___ ___ ___ /___ ___ ___ ___
D D M M M Y Y Y Y

Seizures (problems with…)

1. How often do seizures happen

☐ Never
☐ Almost Never
☐ Sometimes
☐ Often
☐ Almost Always

2. Safety is a problem with seizures

☐ Never
☐ Almost Never
☐ Sometimes
☐ Often
☐ Almost Always

3. Seizures require doctor or hospital visits

☐ Never
☐ Almost Never
☐ Sometimes
☐ Often
☐ Almost Always

4. Seizures result in prolonged recovery (>1 hour)

☐ Never
☐ Almost Never
☐ Sometimes
☐ Often
☐ Almost Always

5. Cause worsening of symptoms (walking, talking)

☐ Never
☐ Almost Never
☐ Sometimes
☐ Often
☐ Almost Always

6. Changed amount of time doing usual activities

☐ Never
☐ Almost Never
☐ Sometimes
☐ Often
☐ Almost Always

Feeding (problems with...) / With G-Tube

7. Significant time to finish meals

- ☐ Never
- ☐ Almost Never
- ☐ Sometimes
- ☐ Often
- ☐ Almost Always

8. Difficulty giving medicine

- ☐ Never
- ☐ Almost Never
- ☐ Sometimes
- ☐ Often
- ☐ Almost Always

9. Not eating enough food)

- ☐ Never
- ☐ Almost Never
- ☐ Sometimes
- ☐ Often
- ☐ Almost Always

10. Choking or difficulty swallowing

- ☐ Never
- ☐ Almost Never
- ☐ Sometimes
- ☐ Often
- ☐ Almost Always

11. Problems with giving meals

- ☐ Never
- ☐ Almost Never
- ☐ Sometimes
- ☐ Often
- ☐ Almost Always

12. Skin problems at G-tube site (irritation, infections...)

- ☐ Never
- ☐ Almost Never
- ☐ Sometimes
- ☐ Often
- ☐ Almost Always

13. Requires support from health care workers

- ☐ Never
- ☐ Almost Never
- ☐ Sometimes
- ☐ Often
- ☐ Almost Always

SLEEP (problems with…)

14. Child sleeps poorly
- ☐ Never
- ☐ Almost Never
- ☐ Sometimes
- ☐ Often
- ☐ Almost Always

15. excessive daytime sleep
- ☐ Never
- ☐ Almost Never
- ☐ Sometimes
- ☐ Often
- ☐ Almost Always

16. Hyperactive at night
- ☐ Never
- ☐ Almost Never
- ☐ Sometimes
- ☐ Often
- ☐ Almost Always

17. Disturbs family sleep
- ☐ Never
- ☐ Almost Never
- ☐ Sometimes
- ☐ Often
- ☐ Almost Always

18. Requires medications for sleep
- ☐ Never
- ☐ Almost Never
- ☐ Sometimes
- ☐ Often
- ☐ Almost Always

BEHAVIOR (problems with…)

19. Affected child in sad mood
- ☐ Never
- ☐ Almost Never
- ☐ Sometimes
- ☐ Often
- ☐ Almost Always

20. Lessened interest in usual activities
- ☐ Never
- ☐ Almost Never
- ☐ Sometimes
- ☐ Often
- ☐ Almost Always

21. Impulsive or unsafe behavior
- ☐ Never
- ☐ Almost Never
- ☐ Sometimes
- ☐ Often
- ☐ Almost Always

22. Aggressive
- ☐ Never
- ☐ Almost Never
- ☐ Sometimes
- ☐ Often
- ☐ Almost Always

23. Repetitive behaviors
- ☐ Never
- ☐ Almost Never
- ☐ Sometimes
- ☐ Often
- ☐ Almost Always

24. Requires medication for help
- ☐ Never
- ☐ Almost Never
- ☐ Sometimes
- ☐ Often
- ☐ Almost Always

DAILY ACTIVITIES

25. Problems with toileting
- ☐ Never
- ☐ Almost Never
- ☐ Sometimes
- ☐ Often
- ☐ Almost Always

26. Appears to be in pain
- ☐ Never
- ☐ Almost Never
- ☐ Sometimes
- ☐ Often
- ☐ Almost Always

27. Contractures/spasms limit activity
- ☐ Never
- ☐ Almost Never
- ☐ Sometimes
- ☐ Often
- ☐ Almost Always

28. Vision impairs activity
- ☐ Never
- ☐ Almost Never
- ☐ Sometimes
- ☐ Often

DESCRIPTION OF THE QUESTIONNAIRE:

Respondents complete EITHER “Feeding – No G-Tube” or “Feeding – With G-tube”, dependent upon feeding status of the patient.

Dimensions	Number of Items	Cluster of Items	Reversed Scoring	Direction of Dimensions
Seizures	6	1-6	1-6	Higher scores indicate better HRQOL.
Feeding – No G-Tube	4	1-4	1-4	
Feeding – With G-Tube	3	1-3	1-3	
Sleep	5	1-5	1-5	
Behaviour	6	1-6	1-6	
Daily Activities	4	1-4	1-4	

SCORING OF DIMENSIONS:

**Item Scaling**	5-point Likert scale from 0 (Never) to 4 (Almost always)
**Weighting of items**	No
**Extension of the Scoring Scale**	Scores are transformed on a scale from 0 to 100.
**Scoring Procedure**	**Step 1: Transform Score**<br>Items are reversed scored and linearly transformed to a 0-100 scale as follows: 0=100, 1=75, 2=50, 3=25, 4=0.<br><br>**Step 2: Calculate Scores**<br>Score by Dimensions:<br>- If more than 50% of the items in the scale are missing, the scale scores should not be computed.<br>- Mean score = Sum of the items over the number of items answered.<br><br>**Total Score**: Sum of all the items over the number of items answered on all the Scales.
**Interpretation and Analysis of Missing Data**	If more than 50% of the items in the scale are missing, the Scale Scores should not be computed.<br><br>If 50% or more items are completed: Impute the mean of the completed items in a scale.
