# Supplementary material for: Psychometric Validation of the CLN2 Quality of Life Questionnaire in Participants with CLN2 Disease Treated with Cerliponase Alfa
Source: Healthcare (Basel). 2024 Nov 8;12(22):2229. doi: 10.3390/healthcare12222229 (PMC11593549; doi:10.3390/healthcare12222229)
Supplement: Supplementary file 1 [file healthcare-12-02229-s001.zip › Supplementary file S2.pdf]

Supplementary file S2

Table S1. Descriptive summary of CLN2 Quality of Life (CLN2 QoL) questionnaire domains at baseline in the BMN 190-201 study

CLN2	N	Mean	Median	Std dev	Min	Max
**Feeding No G-Tube**	22	75.57	81.25	22.57	31.25	100.00
**Seizures**	22	64.20	64.58	18.17	37.50	100.00
**Feeding with G-Tube**	0					
**Sleep**	22	79.77	90.00	25.89	15.00	100.00
**Behaviour**	22	73.67	79.17	13.57	41.67	100.00
**Daily Activities**	22	81.72	87.50	15.35	50.00	100.00
**Total Score**	22	74.20	73.50	13.82	40.00	99.00

Table S2. Descriptive summary Pediatric Quality of Life Inventory (PedsQL) domains at baseline in the BMN 190-201 study

PedsQL	N	Mean	Median	Std dev	Min	Max
**Physical Functioning**	**23**	63.45	62.50	22.15	25.00	100.00
**Emotional Functioning**	22	70.23	70.00	20.67	15.00	100.00
**Social Functioning**	23	49.78	50.00	15.85	25.00	85.00
**Psychosocial Functioning**	23	59.03	57.69	12.40	30.77	82.50
**School Functioning**	22	57.01	58.33	19.64	25.00	100.00
**Total Score**	23	60.73	59.52	12.80	40.48	81.94

Table S3. Descriptive summary Clinical Rating Scale ML and Adapted Clinical Rating Scale scores at baseline in the BMN 190-201 study

	N	Mean	Median	Std dev	Min	Max
Clinical Rating Scale ML	24	3.58	3	1.05	2	6
Adapted Clinical Rating Scale	24	6.50	6	1.18	4	9

Table S4. Ceiling and floor effects for individual items in the CLN2 Quality of Life (CLN2 QoL) questionnaire

**Baseline**				**Ceiling effect**			**Floor effect**		
**Item**	**N**	**Min (%)**	**Max (%)**	**20%**	**35%**	**50%**	**20%**	**35%**	**50%**
How often to seizures happen	22	13.6	4.5	No	No	No	No	No	No
Safety is a problem with seizures	22	31.8	13.6	**Yes**	No	No	No	No	No
Seizures require doctor or hospital visit	22	45.5	4.5	**Yes**	**Yes**	No	No	No	No
Seizures with prolonged recovery	22	36.4	27.3	**Yes**	**Yes**	No	**Yes**	No	No
Caused worsening of symptoms	22	40.9	18.2	**Yes**	**Yes**	No	No	No	No
Changed time doing activities	22	36.4	4.5	**Yes**	**Yes**	No	No	No	No
Significant time to finish meals	22	40.9	22.7	**Yes**	**Yes**	No	**Yes**	No	No
Difficulty giving medicines	22	50	18.2	**Yes**	**Yes**	**Yes**	No	No	No
Not eating enough food	22	54.5	4.5	**Yes**	**Yes**	**Yes**	No	No	No
Choking of difficulty swallowing	22	72.7	4.5	**Yes**	**Yes**	**Yes**	No	No	No
Child sleeps poorly	22	63.6	18.2	**Yes**	**Yes**	**Yes**	No	No	No
Excessive daytime sleep	22	59.1	13.6	**Yes**	**Yes**	**Yes**	No	No	No
Hyperactive at night	22	68.2	9.1	**Yes**	**Yes**	**Yes**	No	No	No
Disturbs family sleep	22	59.1	18.2	**Yes**	**Yes**	**Yes**	No	No	No
Requires medication for help sleep	22	86.4	4.5	**Yes**	**Yes**	**Yes**	No	No	No
Affected child in sad mood	22	40.9	13.6	**Yes**	**Yes**	No	No	No	No
Lessened interested in activities	22	50	4.5	**Yes**	**Yes**	**Yes**	No	No	No
Impulse of unsafe behaviour	22	31.8	9.1	**Yes**	No	No	No	No	No
Aggressive	22	45.5	4.5	**Yes**	**Yes**	No	No	No	No
Repetitive behaviours	22	45.5	22.7	**Yes**	**Yes**	No	**Yes**	No	No
Requires medication for help daily activities	22	86.4	4.5	**Yes**	**Yes**	**Yes**	No	No	No
Problems with toileting	22	38.1	14.3	**Yes**	**Yes**	No	No	No	No
Appears to be in pain	22	68.2	9.1	**Yes**	**Yes**	**Yes**	No	No	No
Contracture/spasm limit activity	22	72.7	13.6	**Yes**	**Yes**	**Yes**	No	No	No
Vision impairs activities	22	81.8	4.5	**Yes**	**Yes**	**Yes**	No	No	No
Requires health workers support	0	No data							
Skin problem at G-tube site	0	No data							
Problems with giving meals	0	No data							
